# Tensor-valued diffusion magnetic resonance imaging in a radiotherapy setting

**DOI:** 10.1016/j.phro.2022.11.005

**Published:** 2022-11-10

**Authors:** Patrik Brynolfsson, Minna Lerner, Pia C. Sundgren, Christian Jamtheim Gustafsson, Markus Nilsson, Filip Szczepankiewicz, Lars E. Olsson

**Affiliations:** a Dept. of Translational Medicine, Division of Medical Radiation Physics, Lund University, Malmö, Sweden; b Random Walk Imaging AB, SE-22002 Lund, Sweden; c NONPI Medical AB, SE-90738 Umeå, Sweden; d Dept. of Hematology, Oncology and Radiation Physics, Skåne University Hospital, Lund, Sweden; e Dept. Diagnostic Radiology, Clinical Sciences Lund, Lund University, Lund, Sweden

**Keywords:** ADC, apparent diffusion coefficient, DIVIDE, Diffusional Variance Decomposition, FA, fractional anisotropy, GTV, gross tumor volume, MD, mean diffusivity, MKA, anisotropic diffusional variance, MKI, isotropic diffusional variance, MRI, magnetic resonance imaging, RT, radiotherapy, SNR, signal to noise ratio, µFA, microscopic fractional anisotropy, MRI in radiotherapy, Tensor-valued diffusion, Brain tumors, Quantitative imaging

## Abstract

**Background and purpose:**

Diagnostic information about cell density variations and microscopic tissue anisotropy can be gained from tensor-valued diffusion magnetic resonance imaging (MRI). These properties of tissue microstructure have the potential to become novel imaging biomarkers for radiotherapy response. However, tensor-valued diffusion encoding is more demanding than conventional encoding, and its compatibility with MR scanners that are dedicated to radiotherapy has not been established. Thus, our aim was to investigate the feasibility of tensor-valued diffusion MRI with radiotherapy dedicated MR equipment.

**Material and methods:**

A tensor-valued diffusion protocol was implemented, and five healthy volunteers were scanned with different resolutions using conventional head coil and radiotherapy coil setup with fixation masks. Signal-to-noise-ratio (SNR) was evaluated to assess the risk of signal bias due to rectified noise floor. We also evaluated the repeatability and reproducibility of the microstructure parameters. One patient with brain metastasis was scanned to investigate the image quality and the transferability of the setup to diseased tissue.

**Results:**

A resolution of 3 × 3 × 3 mm^3^ provided images with SNR > 3 for 93 % of the voxels using radiotherapy coil setup. The parameter maps and repeatability characteristics were comparable to those observed with a conventional head coil. The patient evaluation demonstrated successful parameter analysis also in tumor tissue, with SNR > 3 for 93 % of the voxels.

**Conclusion:**

We demonstrate that tensor-valued diffusion MRI is compatible with radiotherapy fixation masks and coil setup for investigations of microstructure parameters. The reported reproducibility may be used to plan future investigations of imaging biomarkers in brain cancer radiotherapy.

## Highlights

• Demonstrate technical feasibility of tensor-valued diffusion MRI in radiotherapy.
• Diffusion MRI parameters correlate to coil selection and imaging parameters.
• Parameter characteristics are surveyed in healthy brains and one brain tumor.
• Tensor-valued encoding facilitates studies of tumor microstructure in radiotherapy.

## 1 Introduction

The use of magnetic resonance imaging (MRI) in radiotherapy (RT) has increased rapidly in the last decades, through the increased availability of MR scanners in RT departments [1]. The images are primarily used for target delineation and standard follow-up. However, the treatment response in terms of tumor volume changes is delayed by weeks or months, warranting the development of biomarkers that can provide actionable information at an earlier stage [2]. Furthermore, the lack of information on radiosensitivity limits the possibility of individual adjustments to the prescribed RT. At the initial stage, response assessment can be confounded by pseudoprogression (treatment related effects due to radiation, chemo- and immunotherapy), and at later stages, by radionecrosis where healthy tissue appears like residual tumor due to radiation damage [2].

Diffusion-weighted MRI is sensitive to the random motion of water molecules in tissue and provides a promising method for investigations of healthy and diseased tissues [3], [4], [5]. For example, a quantitative measure of the average rate of diffusion is the apparent diffusion coefficient (ADC). The ADC can be used as an imaging biomarker to predict outcome for brain metastases, where tumors that respond well to RT exhibit a higher ADC [6], [7], [8]. By contrast, studies have also found a slight *decrease* in ADC in brain metastases of responding tumors during the same time frame [9]. During and after irradiation there are several processes present in a tumor which can change the ADC in either direction. Cellular injury, apoptosis, and a reduction in tumor cellularity are all expected to yield an elevated ADC, while processes relating to cytotoxic edema, inflammatory cell response, and reduction in tumor blood flow would decrease ADC [10]. Therefore, ADC measurements are considered sensitive but not specific enough to provide detailed information to monitor the tumor during and after treatment.

Recently, a method was introduced to separate the contributions from microscopic diffusion anisotropy and heterogeneous isotropic diffusion by so-called diffusional variance decomposition (DIVIDE) [11], [12], [13]. DIVIDE rely on tensor-valued diffusion encoding, which means that the signal is simultaneously sensitized to diffusion along multiple directions [13]. Szczepankiewicz et al. [11] showed that microscopic anisotropy was related to cell structure eccentricity, and that isotropic heterogeneity was related to cell density variance, features that are not distinguishable by conventional diffusion MRI.

Tensor-valued diffusion encoding has not yet been adapted for RT applications; likely due to the challenges associated with MRI in the RT setup [14]. The main constraint is that patient positioning and geometry must be identical at the time of MRI and RT. This introduces two challenges in the context of MRI. First, the fixation equipment prevents the use of high-performance receiver coils, and second, the fixation equipment and patient geometry often requires that imaging is performed at wide-bore MRI system. Both these factors reduce the hardware performance and ultimately the signal and image quality [15], [16]. For example, external beam irradiation of intracranial tumors requires the use of a fixation mask. This, in turn, prevents the use of conventional head-coil arrays and forces the use of less efficient coils that are placed near the target region without affecting the RT fixation. Finally, the use of a fixation mask during MRI also causes additional discomfort, wherefore the total scan time has a particularly important role for patient comfort.

In order to enable clinical studies of imaging biomarkers, sufficient image quality must be established [17], emphasizing the importance to validate the DIVIDE sequence in the RT workflow. In this study, the aim was to investigate the technical feasibility and performance of tensor-valued diffusion encoding and DIVIDE in an RT setting. To relate the performance to conventional approaches, we deployed a standardized DIVIDE measurement scheme with different receiver coil configurations, and quantified image quality in the brain of healthy volunteers and in a patient with brain metastasis to verify a transferable setup to diseased tissue.

## 2 Material and methods

### 2.1 Diffusional variance decomposition (DIVIDE)

**Fig. 1:**
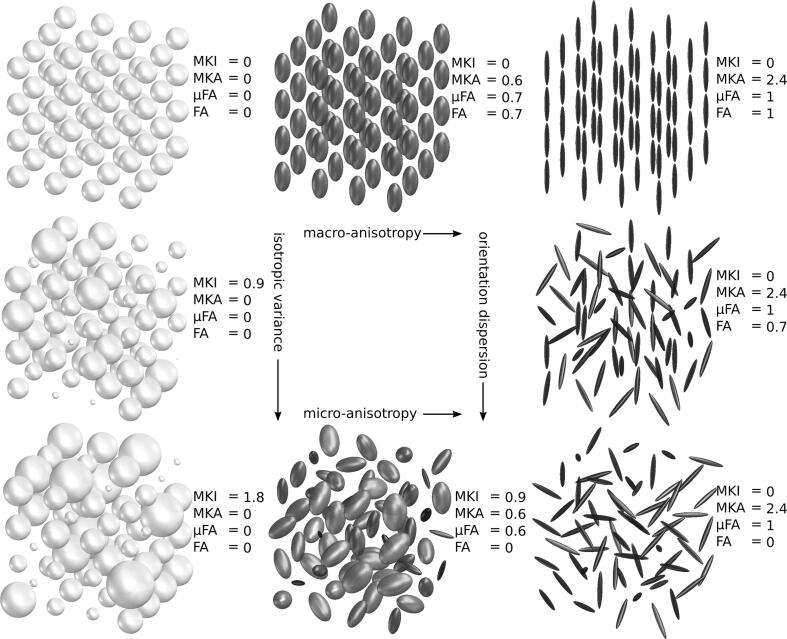
An illustration of how the DIVIDE parameters reflect the underlying diffusion tensor distribution in eight different substrates that have the same mean diffusivity (MD = 1 µm^2^/ms). The first column shows an increase in the variance of isotropic diffusivities (MKI) and the third column shows an increase in orientation dispersion. The first row shows an increase in macroscopic anisotropy (MKA), which affect fractional anisotropy (FA) on the macroscopic and microscopic (µFA) levels. The bottom row shows an increase in microscopic anisotropy with total orientation dispersion, which will leave FA = 0.The figure was adapted and reproduced, with permission, from Szczepankiewicz [18].

DIVIDE distinguishes multiple sources of diffusional variance or diffusional kurtosis. The analysis framework assumes that the diffusion process in tissue can be approximated by a mixture of diffusion tensors where each tensor describes the diffusion in a component of the tissue (Fig. 1) [18], [19]. The term’diffusional variance’ refers to the fact that a single voxel may contain multiple ADC due to diffusion anisotropy, where diffusivity is different across directions, as well as isotropic variance, where the isotropic diffusivity is different across tissue components [11]. DIVIDE allows estimation of the following parameters: mean diffusivity (MD), fractional anisotropy (FA), microscopic fractional anisotropy (μFA), and the diffusional variance caused by isotropic (MKI) and anisotropic diffusion (MKA) [11], [12]. The parameters in this work were calculated in the software dVIEWR powered by MICE Toolkit™ (v. 2021.1.0, Random Walk Imaging AB and NONPI Medical AB, Sweden, https://www.dviewr.com and https://www.micetoolkit.com).

### 2.2 Study subjects

**Fig. 2:**
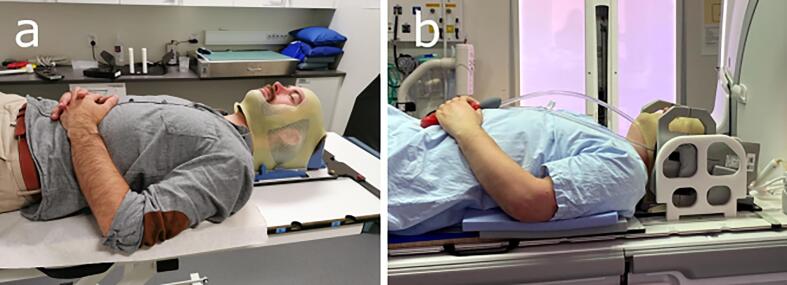
An open fixation mask where the face is not covered (a) and a closed fixation mask positioned with the radiotherapy coil setup (b).

Five healthy volunteers and one patient were included in this study after giving informed consent. The study was approved by the National Ethical Review Board, Sweden (2020–01495). Volunteers were fitted with three-point RT fixation masks by experienced nurses. To improve comfort during the extended MR acquisition time for the volunteers, open-face masks were used (Fig. 2a). The patient, prescribed stereotactic RT (30 Gy, 3 fractions) towards a brain metastasis from primary lung cancer, was examined according to clinical routine using a closed, three-point fixation mask.

### 2.3 MRI acquisition

MRI was performed on a GE Discovery 750w 3 T scanner (software release DV26.0_R03, General Electric, WI, US) with gradient strength 44 mT/m and slew rate 200 T/m/s. To assess the image quality difference due to the RT setup, each volunteer was scanned using two different coil configurations: a 24-channel dedicated head-coil and a 6-channel flex coil in combination with an 8-channel posterior array. Fig. 2b shows a volunteer with fixation mask and flex coils (RT setup).

Tensor-valued diffusion encoding was performed using a 2D spin-echo sequence with echo-planar imaging (sequence prototype supplied by the vendor). The following imaging parameters were used for both coils: acquisition matrix 80 × 80 in 27 contiguous slices, echo time (TE) 119.5 ms, repetition time (TR) 7188 ms, in-plane acceleration factor 2, scan time 9:49 min, b-values of 100, 700, 1400, 2000 s/mm^2^ acquired in 6, 6, 10, 21 directions for linear b-tensor encoding (b_Δ_ = 1) and 6, 6, 10, 15 rotations for spherical b-tensor encoding (b_Δ_ = 0). The gradient waveforms were numerically optimized for the MRI system using the NOW framework (https://github.com/jsjol/NOW) [20] and were compensated for concomitant gradient effects, as described in ref. [21]. The acquisition order of b-tensor shapes and b-values were randomized to reduce effects of heating and systematic signal bias [16], [22]. We aimed to match the image quality in terms of SNR to acquisition protocols reported in Szczepankiewicz et al. [23]. Given the reduced SNR of the 6-channel flex-coil, the voxel size was set to 3 × 3 × 3 mm^3^. Additionally, two subjects were scanned again on a different day. Multiband acceleration can be used to acquire multiple slices simultaneously (SMS), thereby speeding up the acquisition [24]. The technique benefits from having multiple receiver coil elements along the slice direction. However, the RT-coil setup has a relatively small number of coils and may therefore suffer a penalty in performance when combined with a multiband readout. To evaluate the feasibility of SMS with RT-coils a multiband factor of 2 was also used for both coil configurations in one subject. Prior to parameter fitting, Marchenko-Pastur denoising [25] and Gibbs ringing reduction using sub-voxel shifts [26] were applied to the data, and all images were corrected for eddy currents and motion using Elastix [27] with extrapolated target volumes [28]. The analysis was performed using dVIEWR powered by MICE Toolkit™.

### 2.4 Analysis of SNR

We report the fraction of the brain parenchyma where SNR was above 3 and 6 at the highest b-value (b = 2000 s/mm^2^) as a parameter of data quality, denoted Q_3_ and Q_6_ [23]. Since spherical b-tensor encoding was repeated several times for each b-value, SNR was assessed by calculating the voxel-wise ratio of the mean and standard deviation of the signal. To avoid overestimation, SNR was calculated based on data prior to post-processing. We assumed that the noise was approximately Rice distributed, and used a threshold of 3 to identify regions where signal bias was likely to influence signal accuracy [29]. Only voxels within the brain parenchyma were included in the SNR analysis and regions dominated by cerebrospinal fluid were excluded by only considering voxels with MD < 1.5 μm^2^/ms.

### 2.5 Analysis of repeatability and reproducibility

Repeatability was evaluated by acquiring two identical image series consecutively in each coil setup for all subjects, referred to as intra-exam repeatability, similar to previous definition [30]. Additionally, the same imaging protocol was acquired in two subjects on two different days, referred to as inter-exam repeatability. For all subjects, the reproducibility [30] was assessed by comparing the RT-coil setup to the head-coil. The DIVIDE parameters, signal repeatability and reproducibility were assessed by calculating parameter map differences between acquisitions (ΔX = X2 − X1) where ΔX is a distribution of paired voxel-wise differences. The mean and standard deviation of ΔX capture the overall parameter bias and precision. Motion and eddy current corrections were performed with the earliest acquisition as reference. To avoid overestimating the variability due to a misaligned brain periphery and partial-volume effects with cerebrospinal fluid (CSF), only voxels where μFA > 0.7 and MD < 1.5 μm^2^/ms were considered [23]. The repeatability and reproducibility were visualized in Bland-Altman plots and maps of ΔX. The estimated precision pertains to the per-voxel parameter uncertainty; analyzing the average over multiple voxels in a region of interest (ROI) will improve precision.

### 2.6 Patient evaluation

Using the same setup and protocol as for volunteer imaging, MRI examination of the patient was carried out prior to RT on the same scanner as above using the 6-channel flex-coils combined with the 8-channel posterior array. The clinical MR acquisition protocol was scanned with the addition of the tensor-valued diffusion encoding described above.

The gross tumor volume (GTV) was delineated by an experienced radiation oncologist in a contrast enhanced T1-weighted image, according to local clinical routine. SNR for the images with the highest b-value (b = 2000 s/mm^2^) was calculated within the brain parenchyma as well as in the GTV to ensure sufficient image quality.

## 3 Results

### 3.1 Analysis of SNR

**Fig. 3:**
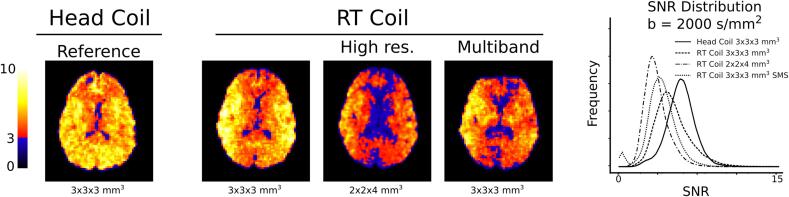
The SNR distribution at b = 2000 s/mm^2^ in one volunteer for the different coils and imaging protocols, where voxels in the blue spectrum exhibits an SNR < 3 and will suffer from signal bias due to the rectified noise floor. The RT-coil SNR maps exhibit low values in the mid-sagittal plane due to the placement and design of the flex coils. Multiband acceleration (SMS) reduced the overall SNR for the RT-coil setup and caused severe distortions in the anterior parts of the image. (For interpretation of the references to color in this figure legend, the reader is referred to the web version of this article.)

The analysis of SNR and its distribution shows that different coil configurations have different performance (Fig. 3 and Fig. S1). As expected, the head-coil leverages the optimal placement of the coil array to produce a homogeneous and high SNR with Q_3_ = 97 % and Q_6_ = 32 %. By contrast, the RT setup showed a markedly lower SNR in the mid-sagittal plane. This is expected since the RT-coils are placed on either side of the head. The larger voxels in the 3 × 3 × 3 mm^3^ configuration exhibited better SNR characteristics with Q_3_ = 93 % and Q_6_ = 23 %, versus Q_3_ = 64 % and Q_6_ = 3 %, for the 2 × 2 × 4 mm^3^ resolution (Fig. S1). Finally, the RT-coils had acceptable performance when combined with multiband acceleration, Q_3_ = 82 % and Q_6_ = 6 % but the image suffered from major distortions, especially in the anterior parts. Considering the SNR performance of the RT-coil, all further comparisons will be done between the head-coil and the RT-coil setup at 3 × 3 × 3 mm^3^ resolution.

### 3.2 Analysis of DIVIDE parameters

**Fig. 4:**
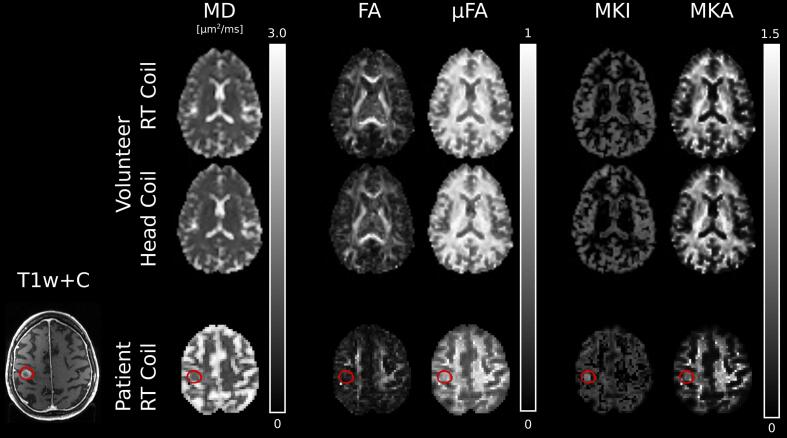
DIVIDE parameter maps from one healthy volunteer in the radiotherapy coil and the head-coil configuration (top two rows) and one patient with a brain metastasis from primary lung cancer (bottom row). The patient row includes a T1-weighted MR image with contrast enhancement from Gadolinium (T1w + C). The gross tumor volume is outlined in red*.* Abbreviations: mean diffusivity (MD), fractional anisotropy (FA), microscopic fractional anisotropy (µFA), isotropic diffusional variance (MKI), anisotropic diffusional variance (MKA), radiotherapy (RT). (For interpretation of the references to color in this figure legend, the reader is referred to the web version of this article.)

**Fig. 5:**
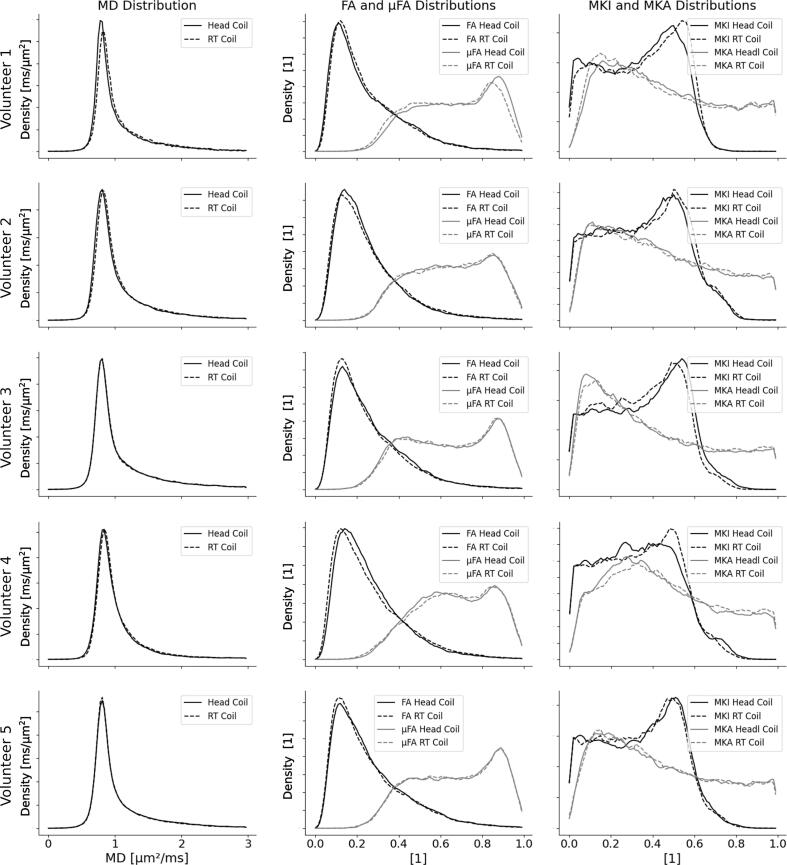
Histograms of parameter values for the RT-coil and the head-coil at a resolution of 3 × 3 × 3 mm^3^ for the healthy volunteers. The three columns show histograms of the mean diffusivity, MD, macroscopic and microscopic fractional anisotropy, FA and µFA, and the distributions of isotropic and anisotropic diffusivities, MKI and MKA.

Parameter maps generated from the head-coil configuration and the RT-coil setup exhibit similar contrast in all investigated parameters, both by visual inspection (Fig. 4) and when investigating the parameter distributions (Fig. 5). The distributions of MD were nearly indistinguishable for the two coils. The distributions of FA, µFA, MKI and MKA showed larger variations between the two coils, but no consistent pattern, or bias, could be seen between the coils.

### 3.3 Analysis of repeatability and reproducibility

**Fig. 6:**
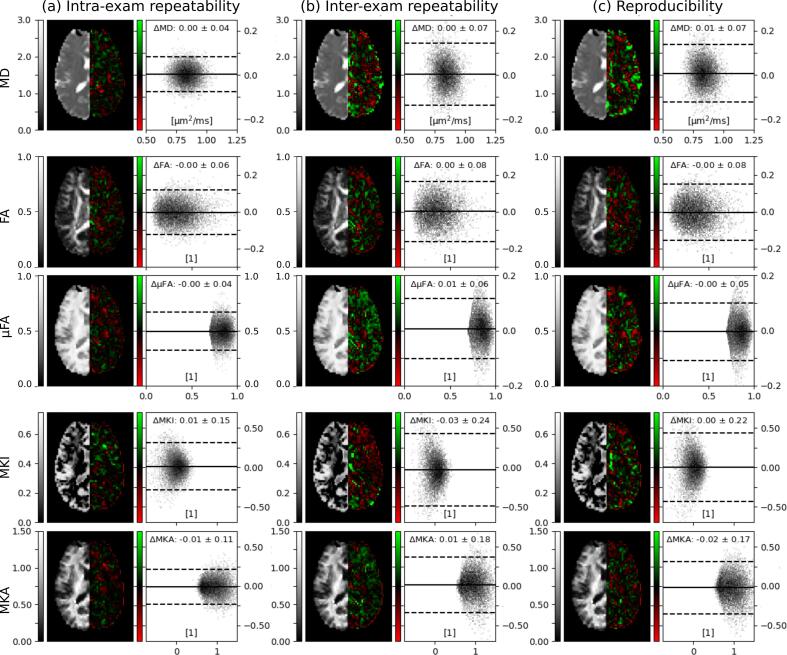
Parameter maps, parameter map differences for repeated scans and analysis of inter- and intra-exam repeatability and reproducibility for volunteer 1. The voxel-wise difference between the first and second acquisition is color-coded in red-green. Bland-Altman plots show the distributions of voxel-wise differences in tissue where μFA > 0.7 and MD < 1.5 μm^2^/ms. Solid and dashed lines show the average and 1.96 standard deviations of the distributions. All configurations showed negligible bias in reproducibility and repeatability of the DIVIDE parameters. Corresponding analyses for all volunteers can be found in Fig. S2-S5. (For interpretation of the references to color in this figure legend, the reader is referred to the web version of this article.)

The evaluation of the intra-exam and inter-exam repeatability measurements and the reproducibility measurements in volunteer 1 showed that bias for all test conditions were negligible, and that the precision was the highest for intra-exam repeatability (Fig. 6). Figs. S2-S5 show corresponding analyses for volunteers 2–5. The resulting bias and variance were very similar across subjects.

### 3.4 Patient example

The SNR levels for the included patient calculated within the brain parenchyma were Q_3_ = 88 % and Q_6_ = 16 %. The patient had a brain metastasis located in the right frontal lobe with a GTV of 2.5 cc (90 voxels). Median SNR ± 1 standard deviation within the GTV was 5.5 ± 1.4. Q_3_ and Q_6_ were 93 % and 34 %, respectively, which is in line with healthy volunteers. DIVIDE parameter maps were successfully generated (Fig. 4).

## 4 Discussion

Our study has demonstrated the feasibility of tensor-valued diffusion encoding on an MR-scanner dedicated for RT with flex-coils that enable imaging with fixation masks. The technical evaluation demonstrated sufficient SNR at b = 2000 s/mm^2^ at an isotropic image resolution of 3 mm. The positions of coil elements in the RT-coil configuration produced spatial SNR variations, with a pronounced reduction in SNR in the mid-sagittal plane, which is not observed when using the head-coil. Nevertheless, repeatability and reproducibility measurements showed that the RT setup and the conventional head-coil setup exhibit similar repeatability characteristics. Our results agree with Szczepankiewicz et al [23], who reported similar values of Q_3_, Q_6_ and voxel-wise test–retest distributions in a conventional imaging setup with a resolution of 2 × 2 × 4 mm^3^.

The MR scanners used in RT departments generally have a lower gradient performance due to a wider bore compared to diagnostic MR scanners, which causes longer diffusion encoding and echo times for any given b-value [23]. Further, due to the reduced coil coverage and number of receiver coils of the present 6-channel flex-coil setup, the SNR was reduced compared to the head-coil setup. However, a trade-off can be made between SNR, resolution, and scan time. Sufficient SNR is crucial, as a low SNR may introduce parameter bias, which has been shown most prominent for MKI [23]. Both precision and accuracy in all DIVIDE parameters have previously been shown, through simulations, to improve with increasing SNR [23]. In this study, SNR was increased for the RT-coil setup by increasing the voxel size from 2 × 2 × 4 mm^3^ to 3 × 3 × 3 mm^3^, as scanning time had to be kept at a minimum. While we decreased the in-plane resolution, we increased the through-plane resolution, as well as achieved isotropic voxels and increased the SNR. We found this to be a reasonable trade-off for current investigation. SNR distributions with Q_3_ around 90 % at b = 2000 s/mm^2^ were demonstrated in both healthy tissue for the volunteers and in tumor tissue for the patient. Nevertheless, there may exist areas with insufficient SNR, especially in the central parts of the brain and deep gray matter. Hence, SNR analysis will be important to estimate the validity of the DIVIDE analysis in any future patient studies Since dMRI has inherently low SNR, the spatial resolution is often—if not always—below that of sequences tailored to morphological imaging. Although methods like DIVIDE can resolve sub-voxel heterogeneity, the low resolution does limit its applicability in studies of small lesions. Nevertheless, the SNR can be vastly improved at MRI systems with higher gradient strength due to the reduced TE [23], and we predict that the gradient waveforms can be further optimized to improve SNR even at the currently used gradient strength [20].

The bias in MD and FA introduced when using two different receive coils has been investigated previously by analyzing images from 8-channel and 32-channel head-coils [31]. The study indicated that parameter maps from data acquired using different coils must be interpreted with caution or if possible, avoided. We observed some minor differences comparing histograms between the two coils for the DIVIDE parameter estimations. Therefore, to avoid potential coil bias in clinical trials, the same receive coil should be used throughout longitudinal measurements and follow-ups. Our analysis showed a negligible bias in all test–retest scenarios, and a higher precision in the intra-exam repeatability scenario.

The RT-coil setup was not suitable for multiband acceleration (Fig. 3), partly due to insufficient coil coverage in the cranio-caudal direction resulting in lower SNR. However, severe susceptibility-induced distortion in the anterior part of the brain was the main reason for not using multiband acceleration. In the RT setting, where scan time is an important parameter due to patient discomfort, the scanned volume should be limited, since acquisition time increases with the number of acquired slices. Although we do not obtain whole brain coverage with the current parameters, the 8 cm slab is sufficient to include one or several tumors depending on patient specific conditions.

Previous work investigating the DIVIDE imaging technique in brain tumors, highlight the possibility to measure microscopic properties using the DIVIDE technique, and the potential to find novel biomarkers for treatment response [11], [32], [33]. Merely using ADC as marker for treatment response may miss fundamental changes in the tissue, as illustrated in Fig. 1, where all voxels have identical ADC. The patient example in this study demonstrates successful DIVIDE analysis which paves the way for further studies, investigating the microstructure of brain tumors and how the DIVIDE parameter maps may correspond with the effects of RT.

We acknowledge the following study limitations. Firstly, although we performed SNR estimation on data prior to any correction or processing, the estimation may be influenced by the image reconstruction as well as subject motion in the head-coil setup. A positive SNR bias means that a threshold of SNR = 3 may be insufficient to avoid bias in the signal and estimated DIVIDE parameters. Secondly, we did not correct for geometric distortions induced by susceptibility effects. Susceptibility-induced distortions depend on the readout and are therefore the same for tensor-valued and conventional diffusion encoding, which are currently used to aid RT planning and clinical follow-up [34]. Further, we note that image distortions due to non-linear gradients were corrected in the vendor image reconstruction, although, effects on the diffusion encoding were not. However, we expect the effects of gradient non-linearity on both spatial and diffusion encoding to be negligible as the brain tissue is close to the isocenter. For methods that require imaging further from the isocenter, several correction approaches can be used [35], [36], [37]. Finally, the analysis assumes that the heterogeneous diffusion in each voxel is approximately multi-Gaussian, i.e., we assume that diffusion-time effects are negligible [16]. This assumption may be violated in tumor tissue, and thereby cause a bias in the estimated parameters [16], [38], [39]. The size and relevance of this effect will be the subject of future studies.

In conclusion, this is the first time DIVIDE is applied in the RT setting. A technical validation is the first block in the imaging biomarker roadmap suggested by O’Connor et al [17]. Hence, the result of this study is a pre-requisite to initiate the investigation of DIVIDE parameters as potential imaging biomarkers for early treatment response. The additional information the DIVIDE parameters provide compared to ADC and currently existing image sequences, potentially enables new possibilities for individual adaptation of RT which will be of large interest to explore in future studies.

## Declaration of Competing Interest

The authors declare the following financial interests/personal relationships which may be considered as potential competing interests: P.B. is a shareholder in NONPi Medical AB, developer of dVIEWR and MICE Toolkit. F.S. and M.N. are shareholders in Random Walk Imaging AB and are inventors on patents related to the topic. The other authors have nothing to declare.
