# Supplementary material for: Tensor-valued diffusion magnetic resonance imaging in a radiotherapy setting
Source: Phys Imaging Radiat Oncol. 2022 Nov 10;24:144–51. doi: 10.1016/j.phro.2022.11.005 (PMC9679029; doi:10.1016/j.phro.2022.11.005)
Supplement: Supplementary data 1 [file mmc1.docx]

**Supplementary material**





**Figure S1.** Signal-to-noise ratios (SNR) displayed for each volunteer and coil configuration, with the volume of the brain mask (y-axis) fulfilling SNR levels (x-axis) as indicated by the plot. The black and gray lines represent the radiotherapy (RT) coil setup and the head coil, respectively. All volunteers were evaluated with the resolution 3x3x3 mm^3^. In addition, volunteer 1 was evaluated with the resolution 2x2x4 mm^3^ as well as the 3x3x3 mm^3^ using multiband acceleration (SMS) for each coil configuration. The two dotted vertical lines mark SNR=3 (Q_3_) and SNR=6 (Q_6_).


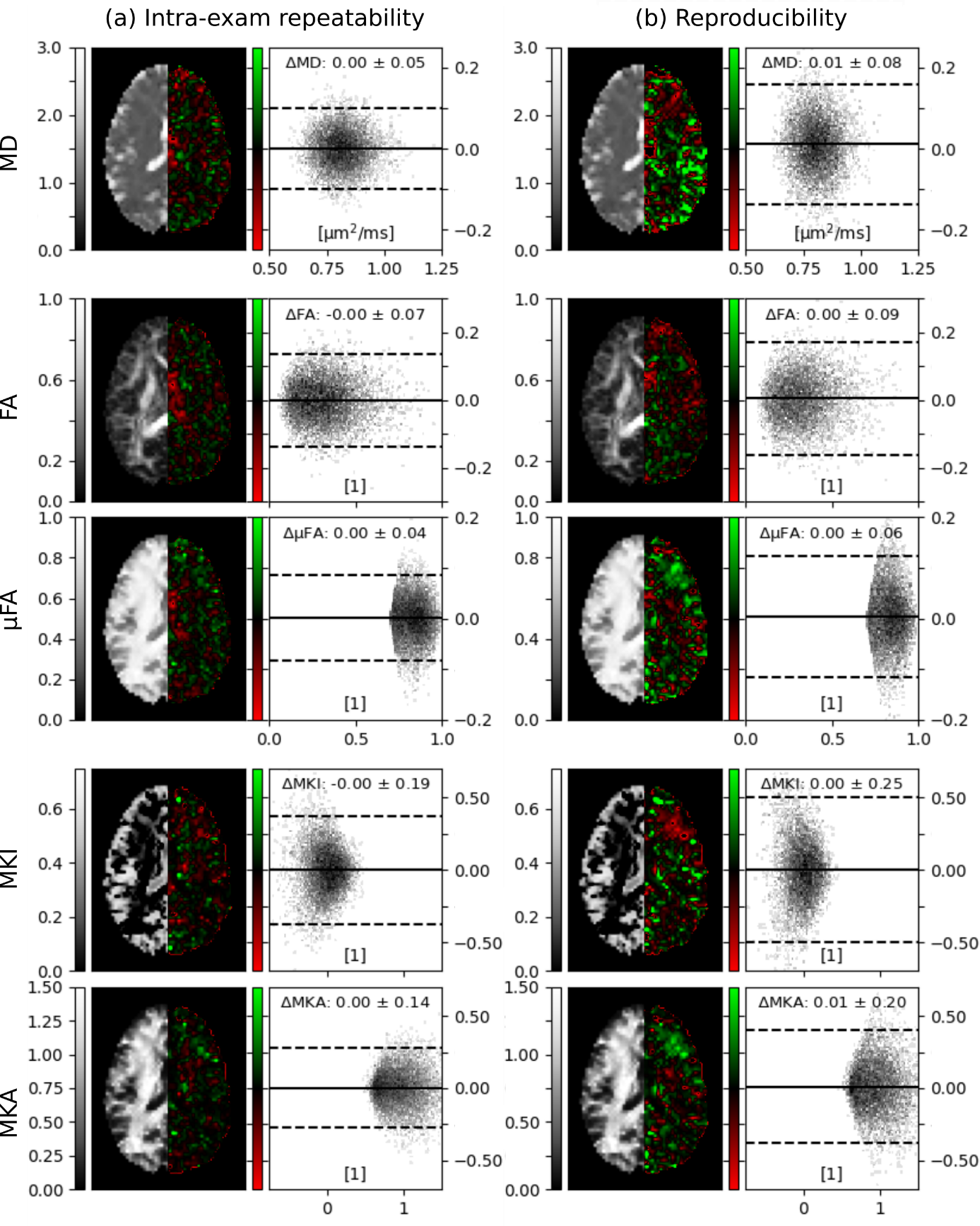


**Figure S2.** Parameter maps, parameter map differences for repeated scans and analysis of intra-exam repeatability and reproducibility for volunteer 2. The voxel-wise difference between the first and second acquisition is color-coded in red-green. Bland-Altman plots show the distributions of voxel-wise differences in tissue where μFA>0.7 and MD<1.5 μm^2^/ms. Solid and dashed lines show the average and 1.96 standard deviations of the distributions. All configurations showed negligible bias in reproducibility and repeatability of the DIVIDE parameters.


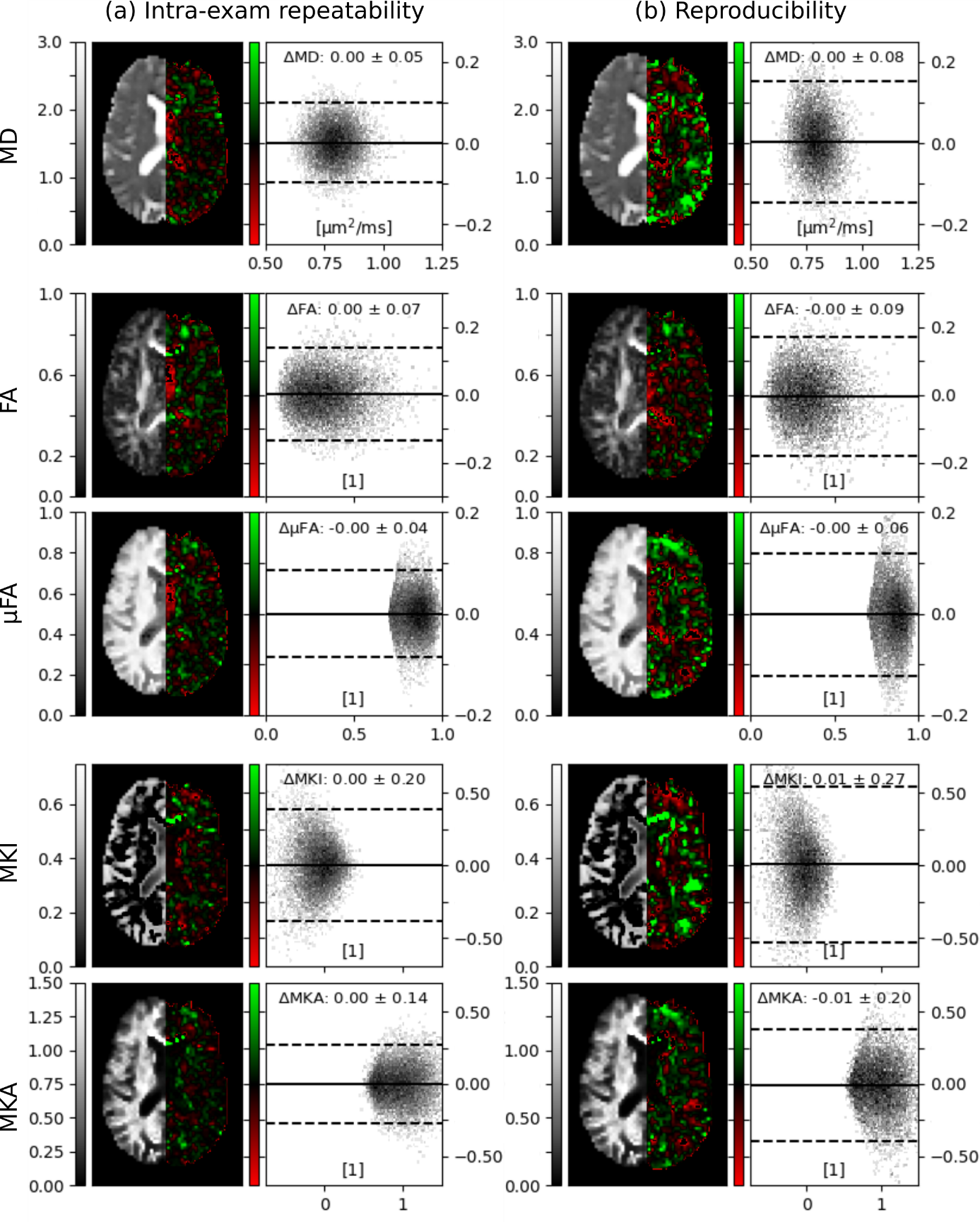


**Figure S3.** Parameter maps, parameter map differences for repeated scans and analysis of intra-exam repeatability and reproducibility for volunteer 3. The voxel-wise difference between the first and second acquisition is color-coded in red-green. Bland-Altman plots show the distributions of voxel-wise differences in tissue where μFA>0.7 and MD<1.5 μm^2^/ms. Solid and dashed lines show the average and 1.96 standard deviations of the distributions. All configurations showed negligible bias in reproducibility and repeatability of the DIVIDE parameters.


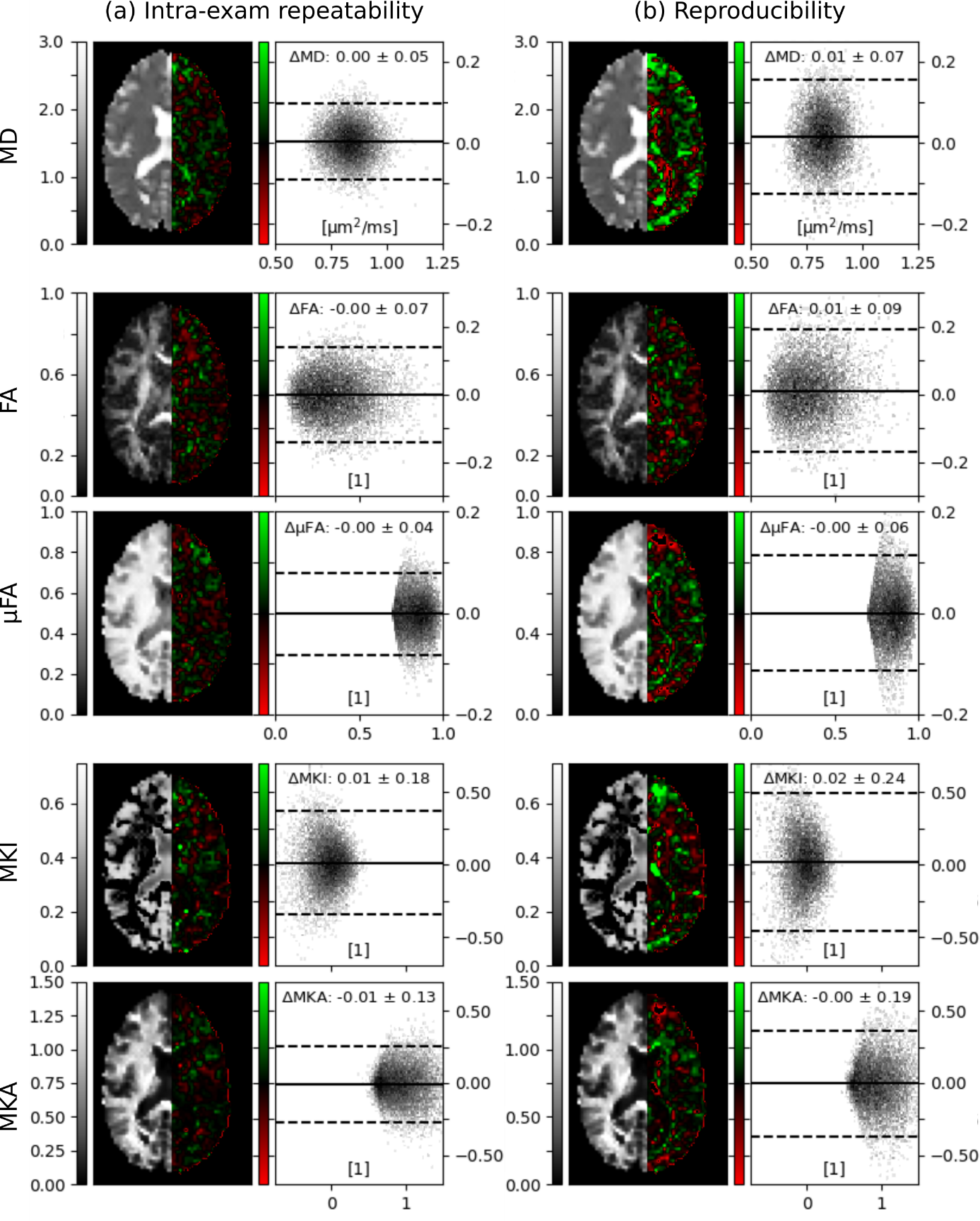


**Figure S4.** Parameter maps, parameter map differences for repeated scans and analysis of intra-exam repeatability and reproducibility for volunteer 4. The voxel-wise difference between the first and second acquisition is color-coded in red-green. Bland-Altman plots show the distributions of voxel-wise differences in tissue where μFA>0.7 and MD<1.5 μm^2^/ms. Solid and dashed lines show the average and 1.96 standard deviations of the distributions. All configurations showed negligible bias in reproducibility and repeatability of the DIVIDE parameters.


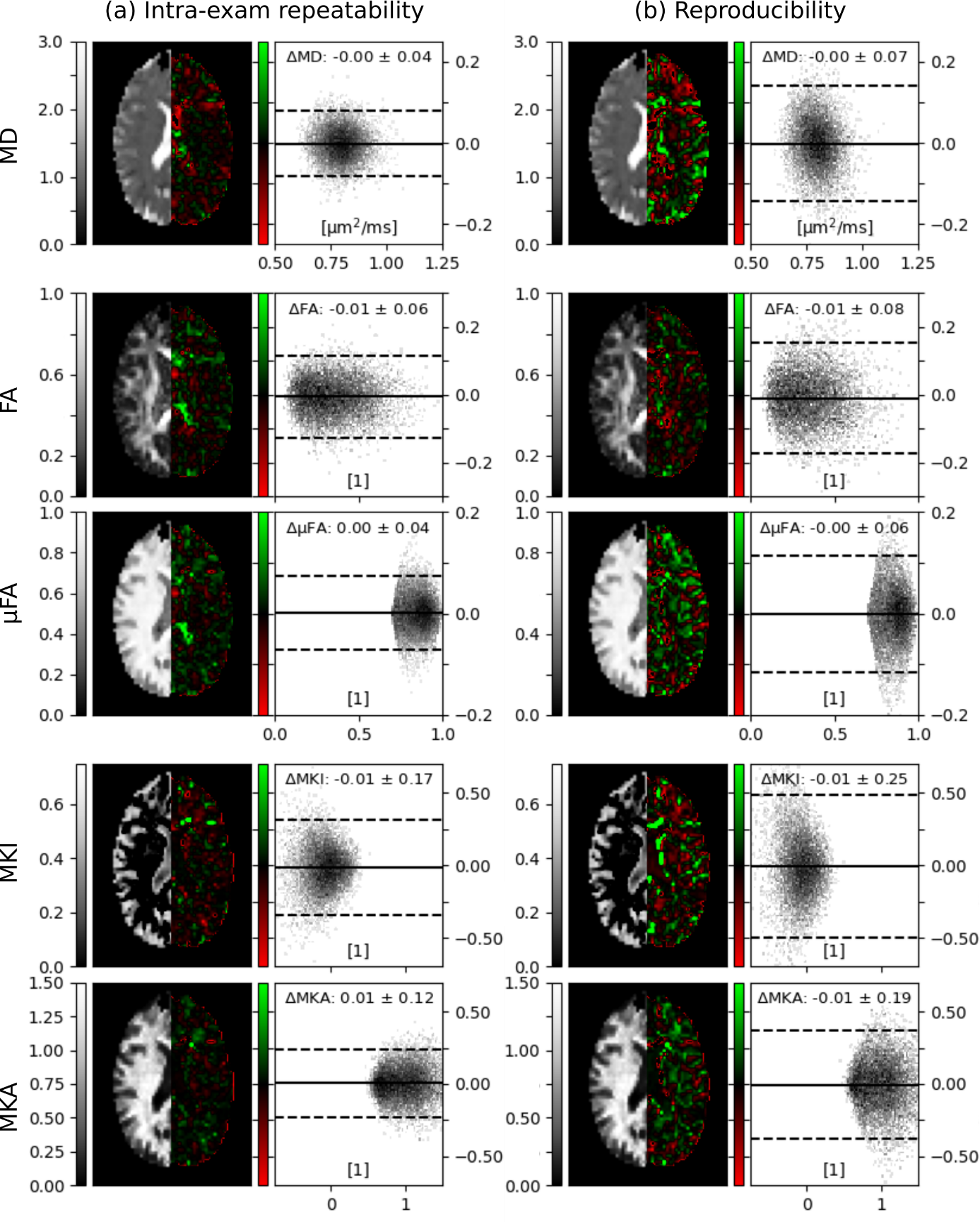


**Figure S5.** Parameter maps, parameter map differences for repeated scans and analysis of intra-exam repeatability and reproducibility for volunteer 5. The voxel-wise difference between the first and second acquisition is color-coded in red-green. Bland-Altman plots show the distributions of voxel-wise differences in tissue where μFA>0.7 and MD<1.5 μm^2^/ms. Solid and dashed lines show the average and 1.96 standard deviations of the distributions. All configurations showed negligible bias in reproducibility and repeatability of the DIVIDE parameters.
